# Establishment of replication-competent vesicular stomatitis virus-based recombinant viruses suitable for SARS-CoV-2 entry and neutralization assays

**DOI:** 10.1080/22221751.2020.1830715

**Published:** 2020-10-17

**Authors:** Hongyue Li, Chaoyue Zhao, Yuhang Zhang, Fei Yuan, Qi Zhang, Xuanling Shi, Linqi Zhang, Chengfeng Qin, Aihua Zheng

**Affiliations:** a State Key Laboratory of Integrated Management of Pest Insects and Rodents, Institute of Zoology, Chinese Academy of Sciences, Beijing, People’s Republic of China; b CAS Center for Excellence in Biotic Interactions, University of Chinese Academy of Sciences, Beijing, People’s Republic of China; c CAS Key Laboratory of Pathogenic Microbiology and Immunology, Institute of Microbiology, Chinese Academy of Sciences, Beijing, People’s Republic of China; d Center for Global Health and Infectious Diseases, Comprehensive AIDS Research Center, and Beijing Advanced Innovation Center for Structural Biology, School of Medicine, Tsinghua University, Beijing, People’s Republic of China; e Department of Virology, State Key Laboratory of Pathogen and Biosecurity, Institute of Microbiology and Epidemiology, Academy of Military Medical Sciences, Beijing, People’s Republic of China; f Key Laboratory of Tropical Translational Medicine of Ministry of Education, School of Tropical Medicine and Laboratory Medicine, Hainan Medical University, Haikou, People’s Republic of China

**Keywords:** SARS-CoV-2, VSV, replication-competent, neutralization assay, entry

## Abstract

Replication-competent vesicular stomatitis virus (VSV)-based recombinant viruses are useful tools for studying emerging and highly pathogenic enveloped viruses in level 2 biosafety facilities. Here, we used a replication-competent recombinant VSVs (rVSVs) encoding the spike (S) protein of SARS-CoV-2 in place of the original G glycoprotein (rVSV-eGFP-SARS-CoV-2) to develop a high-throughput entry assay for SARS-CoV-2. The S protein was incorporated into the recovered rVSV-eGFP-SARS-CoV-2 particles, which could be neutralized by sera from convalescent COVID-19 patients. The recombinant SARS-CoV-2 also displayed entry characteristics similar to the wild type virus, such as cell tropism and pH-dependence. The neutralizing titers of antibodies and sera measured by rVSV-eGFP-SARS-CoV-2 were highly correlated with those measured by wild-type viruses or pseudoviruses. Therefore, this is a safe and convenient screening tool for SARS-CoV-2, and it may promote the development of COVID-19 vaccines and therapeutics.

## Introduction

COVID-19 is a life-threatening disease caused by coronavirus SARS-COV-2 [1,2]. More than eight million SARS-CoV-2 infections and 435,619 deaths have been reported, worldwide, from December 8, 2019 to June 16, 2020, by the World Health Organization (WHO). Effective vaccines or therapeutics are currently unavailable. SARS-CoV-2 belongs to the coronavirus family. It is an enveloped virus with three structural proteins, including spike (S) protein, membrane (M) protein, and envelope (E) protein [3]. The S protein is responsible for receptor attachment and membrane fusion during entry into target cells. This protein is further processed into S1 and S2 by serine protease TMPRSS2 [4–6]. During infection, the S protein is the major target of humoral and cellular immune responses in the host [7]. Consequently, the S protein is the most important target for vaccines and therapeutics.

VSV is a bullet-shaped, negative-strand RNA virus in the *Rhabdoviridae* family [8]. It has five structural genes, which encode N (nucleocapsid), P (phosphoprotein), M (matrix protein), G (glycoprotein), and L (large protein). The genome of VSV is simple and easy to manipulate [9]. VSV can tolerate and package foreign envelope proteins on its surface, which makes VSV an ideal tool for studying the function of heterologous glycoproteins from other viral pathogens as a form of recombinant virus [10–12].

In this report, we describe the generation and characterization of a replication-competent VSV virus that expresses SARS-CoV-2 spike (S) protein. The recombinant virus was able to infect SARS-CoV-2 susceptible cells. The infection could be neutralized using sera from COVID-19 convalescent patients and monoclonal antibodies targeting the S protein. Levels of neutralizing activity based on the replication-competent VSV system strongly correlated with levels from both pseudotype and live virus systems. Therefore, replication-competent rVSV is a promising tool to study SARS-CoV-2 entry process and immune responses to vaccines.

## Materials and methods

### Ethics statement

The rVSV studies were conducted under biosafety level 2 (BSL2) conditions. Research with live SARS-CoV-2 was performed in a biosafety level 3 (BSL3) facility. Human serum samples from COVID-19 convalescent patients were obtained from the Department of Virology, State Key Laboratory of Pathogen and Biosecurity, Institute of Microbiology and Epidemiology, Academy of Military Medical Sciences, Beijing, China. A written consent form was obtained from the patients before they participated in this study. Serum samples were incubated at 56°C for 30 min to inactivate potential viruses.

### Cells, antibodies, and proteins

Vero cells (American Type Culture Collection [ATCC], CCL-81), HEK293T (ATCC, CRL-3216), BHK-21 (ATCC, CCL-10), ST (ATCC, CRL-1746), PK15 (ATCC, CCL-33), A549 (ATCC, CCL-185), HeLa (ATCC, CCL-2), MDBK (ATCC, CCL-22), and Huh7.5 (human hepatocarcinoma cells) were maintained in Dulbecco’s modified Eagle’s medium (DMEM) supplemented with 10% fetal bovine serum (FBS), 1% L-glutamine, and 1% penicillin–streptomycin. All cells were incubated at 37°C with 5% CO_2_. Rabbit anti-SARS-CoV-2 S-RBD polyclonal antibody (Cat. 40150-RP01) and SARS-CoV-2 Spike RBD domain (Cat: 40592-V08B-B) were purchased from Sino Biological Inc. (Beijing, China).

### Construction, rescue, and characterization of rVSV viruses

The rVSV vector was designed, synthesized, and constructed as described previously [9]. The eGFP encoding sequences were added at nt62 to generate the rVSV-eGFP-G plasmid, which was used as the backbone for other plasmids. The humanized spike protein coding sequence of SARS-CoV-2 Wuhan-Hu-1 strain (GeneBank: YP_009724390.1) was synthesized by Genewiz Suzhou and inserted between MluI and NotI sites into the rVSV-eGFP-G plasmid. The leader sequence of the S coding sequence was the original signal peptide. Primers used to amplify the spike gene were 5'-gtttccttgacacgcgtaccatgttcgtgttcctcgtgc-3’ and 5'-gtgcagggcggccgctcaggtgtagtggagcttcacgc-3’. The resulting backbone plasmid was named rVSV-eGFP-SARS-CoV-2, with the VSV glycoprotein coding sequence (3845–5380) being replaced by that of the SARS-CoV-2 spike protein. The rVSVs were rescued using a reverse genetics approach [13]. Briefly, HEK293T cells were transfected with 1.6 µg of rVSV backbone plasmid and five supporting plasmids encoding T7 polymerase (8.1 µg), N (1.286 µg), P (639 ng), M (169.9 ng), and L (169.9 ng) of VSV using the calcium phosphate method. Recovery of the viruses was determined by cytopathic effects and eGFP expression. Viruses in the supernatant were harvested and passaged on Vero cells to obtain virus stocks. The rVSVs were purified by one step ultracentrifugation at 35,000 rpm (SW41 rotor, Beckman, Fullerton, CA, USA) at 4°C for 2 h. The pellets containing purified viral particles were resuspended with PBS. Expression of spike protein in infected cells and on purified viral particles was verified by western blot analysis using polyclonal antibodies against the RBD domain of SARS-CoV-2 S.

### rVSV infection and neutralization assay

A focus-forming assay was performed in Vero cells to titrate the viral titers. Cells were seeded in 96-well plates at 1.5 × 10^4^ cells/well in triplicate 24 h before infection. The viruses were serially diluted ten-fold in DMEM with 2% FBS. After the removal of culture media, diluted viral solution was added to the cells. Three hours later, the cells were washed once and incubated with DMEM containing 2% FBS and 20 mM NH_4_Cl at 28°C. GFP positive cell numbers were counted 12–24 h post-infection using a fluorescent microscope. Wells with around 100 GFP foci were counted to calculate the titer. Viral titer was expressed as focus-forming units per ml (FFU/ml).

For the plaque-forming assay, Vero cells were seeded in 24-well plate at 8 × 10^4^ cells/well, in triplicate, 24 h before infection. The viral samples were serially diluted ten-fold in DMEM with 2% FBS. After the removal of culture media, diluted viral solution was added to the cells. After three hours, the cells were washed once with PBS and incubated with DMEM containing 2% FBS and 2% CMC-Hanks at 28°C to allow plaques to form. The media were aspirated, and the cells were stained with crystal violet 3 d later.

For the focus reduction neutralization test (FRNT), 100 FFU of rVSVs were incubated with five-fold serially diluted, heat-inactivated sera, recombinant protein or mAbs at room temperature for 30 min. The mixtures were then layered onto cells in 96-well plates. Three hours later, culture media were removed, and fresh DMEM containing 2% FBS and 20 mM NH_4_Cl was added. GFP positive cells were counted 20 h after infection using a fluorescent microscope or Opera Phenix High Content Screening System (PerkinElmer, Waltham, MA, USA). Neutralizing titers were calculated as 50% inhibition of virus infection (IC_50_) using the Reed-Muench method.

For the pH-dependent endocytosis experiment, Vero cells were seeded in 96-well plates at 1.5 × 10^4^ cells/well in triplicate. After 18–24 h, the viruses were diluted in DMEM containing 2% FBS and various concentration of NH_4_Cl (0–20 mM), and incubated with Vero cells at 37°C. Three hours later, the cells were washed once and incubated with DMEM containing 2% FBS and 20 mM NH_4_Cl at 37°C. Viral titers were determined 20 h post-infection as described above.

### Growth curve of rVSVs

Vero cells (4 × 10^6^) in a T75 flask was incubated with rVSVs at an MOI of 0.01. After three hours, the culture medium was replaced with DMEM plus 2% FBS. The cells were grown at 28°C, and the supernatant was harvested every 12 h. Titration was performed using a focus-forming assay.

### Pseudovirus preparation and neutralization assay

VSV-based SARS-CoV-2 pseudovirus with a luciferase reporter was obtained from division of HIV/AIDS and sex-transmitted virus vaccines, Institute for Biological Product Control, National Institutes for Food and Drug Control (NIFDC), Beijing, People’s Republic of China [14]. Briefly, BHK-21 cells were infected by recombinant vaccinia virus expressing T7 RNA polymerase and co-transfected with pVSV-ΔG-luciferase plasmid and four supporting plasmids encoding N, P, G, L at a ratio of 5:3:5:8:1. This was followed by three passages of the primary recovery supernatant on cells transfected with pCAGGS-G to generate the working stocks of G*ΔG-VSV [15]. HEK293T cells transfected with SARS-CoV-2 S expressing plasmid were infected by G*ΔG-VSV 24 h post transfection. The pseudoviruses containing supernatant were harvested 24 h post transfection after infection [14].

For the pseudovirus based neutralization assay (PBNA), the pseudoviruses were incubated with a three-fold serial dilution of samples, in triplicate, for 1 h at 37°C. Then the mixtures were loaded to Vero cells at ∼90% confluency in a 96-well plate and incubated at 37°C for 24 h in a 5% CO_2_ environment. The luminescence was measured as previously described [16]. The IC_50_ values were calculated using Reed-Muench method.

### SARS-CoV-2 neutralization assay

SARS-CoV-2 plaque reduction neutralization test (PRNT) was performed in a certified BSL3 laboratory. Vero cells were seeded in 24-well plates and reached ∼90% confluence at the point of infection. Sera from COVID-19 patients were heat-inactivated at 56°C for 30 min. Three-fold serial dilutions of sera were mixed with equal volumes of viral solution to a final concentration of 200 PFU/ml and incubated at 37°C for 1 h. The virus-serum mixtures (250 µl/well) were loaded on 24-well plates and incubated at 37°C for 1 h. Then, the cells were overlaid with 1% low-melting point (LMP) agarose in DMEM with 2% FBS. At 2 d post-infection, the cells were fixed with 4% formaldehyde and stained with 0.2% crystal violet to visualize the plaques.

## Results

### Rescue and characterization of replication-competent rVSV expressing SARS-CoV-2 S protein

**Figure 1.**
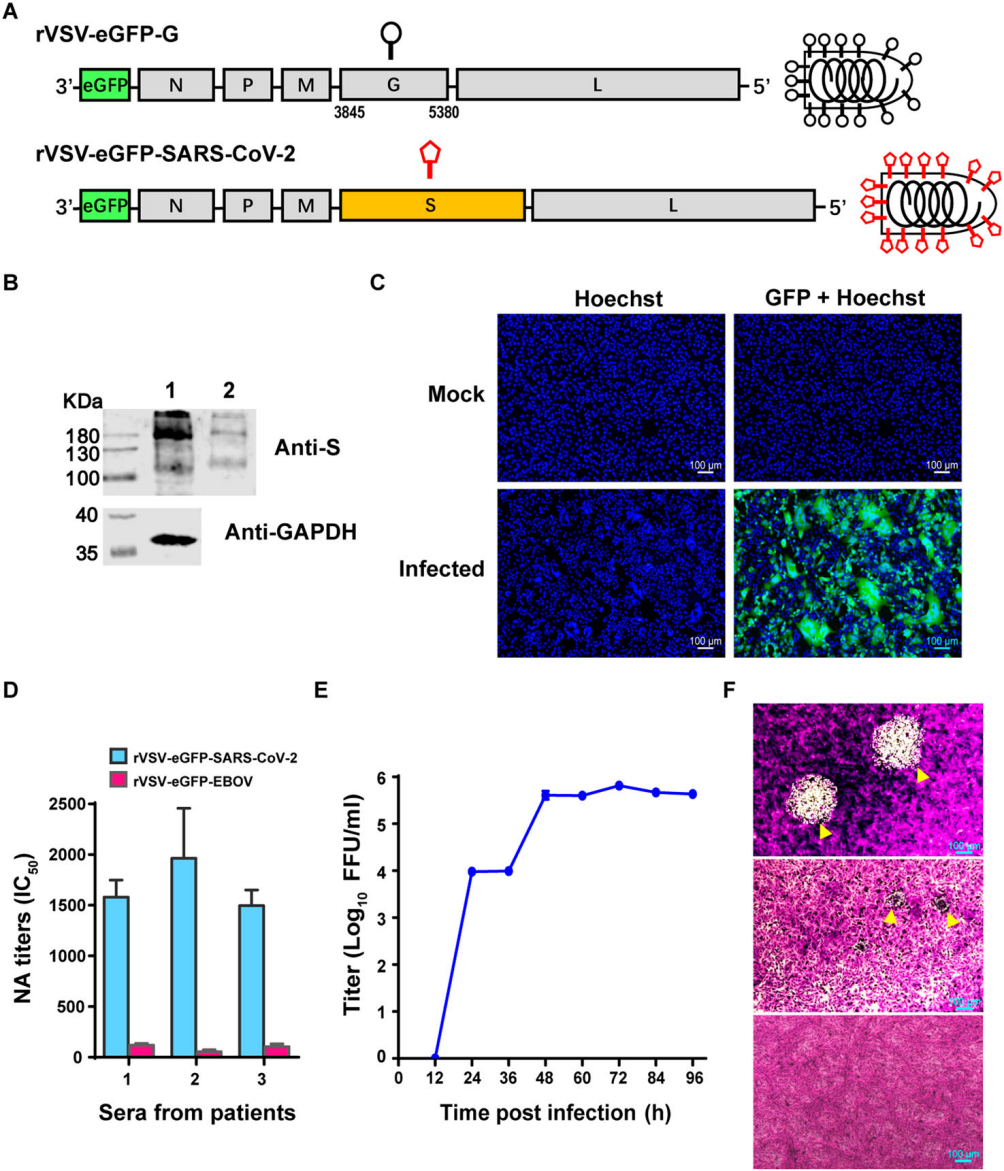
Characterization of rVSV-eGFP-SARS-CoV-2. (A) Schematic diagrams showing genome organization in rVSV vectors. The original VSV G gene was replaced by SARS-CoV-2 S gene to generate rVSV-eGFP-SARS-CoV-2. eGFP was inserted into the first position of the genome. (B) Lysate of rVSV-eGFP-SARS-CoV-2 producing cells (Lane 1) and purified rVSV-eGFP-SARS-CoV-2 (Lane 2) were analysed by western blot using an antibody recognizing the RBD domain of the S protein. (C) Images of mock- (upper panels) or rVSV-eGFP-SARS-CoV-2-infected (lower panels) Vero cells stained with Hoechst to label the nuclei (blue). The GFP signal is shown in green. The overlay of blue and green is shown to visualize syncytia. (D) The titers of neutralizing antibodies (NAbs) in three serum samples from COVID-19 convalescent patients were determined against rVSV-eGFP-SARS-CoV-2 by a focus reduction neutralization test. rVSV-eGFP-EBOV (Ebola virus) were used as a control virus. (E) Growth kinetics of rVSV-eGFP-SARS-CoV-2 in Vero cells (MOI=0.0l). Viral titers were measured by a focus-forming assay and expressed as focus forming units per ml (FFU/ml). Error bars in (D-E) indicate standard deviation of the mean (n=3). (F) Plaque morphology of rVSVs in Vero cells. The plaques of rVSV-eGFP-EBOV (upper panel) or rVSV-eGFP-SARS-CoV-2 (middle panel) at 72 h post-infection indicated by yellow arrowhead were visualized under microscope. Lower panel: Mock-infected cells (control). The data represent three independent experiments.

To generate replication-competent rVSV, the original G protein-encoding gene was replaced by the SARS-CoV-2 WH01 stain S protein gene. An eGFP reporter was inserted in front of the N gene to facilitate viral detection (Figure 1A). The resulting rVSV-eGFP-SARS-CoV-2 was recovered using established methods [13]. Since VSV is an attenuated viral vector, the manipulations of rVSVs were conducted in a BSL2 laboratory. We purified the viral particles from the cell culture supernatant after three passages by ultracentrifugation, and expression of SARS-CoV-2 S protein was examined by western blot. Two bands: 190 and 110 kDa, respectively, representing S and S1, appeared in both virions and virus-infected cell lysates (Figure 1B). During rVSV infection, infected Vero cells formed syncytia, demonstrating that spike protein expression on the plasma membrane induced cell–cell fusion (Figure 1C). To evaluate whether S protein expressed by rVSV displayed proper conformation with major immunogenic epitopes exposed, a focus reduction neutralization test (FRNT) was performed using serum samples from COVID-19 convalescent patients. The neutralizing antibody (NAb) titers in sera from COVID-19 patients ranged from 1497 to 1963, while no neutralizing activities were detected towards rVSV-eGFP-EBOV (Ebola virus) (Figure 1D). These results indicated that SARS-CoV-2 spike protein expressed by rVSV displayed neutralizing epitopes.

The growth curve of rVSV-eGFP-SARS-CoV-2 was determined in Vero cells at an MOI of 0.01. The supernatant containing viral particles was collected at multiple time points up to 96 h, and the virus titers were measured by focus forming assay. The rVSV-eGFP-SARS-CoV-2 reached the maximum titer of 5.8 × 10^5^ focus forming unit/ml (FFU/ml) at 72 h post-infection (Figure 1E). We compared the plaque formation between rVSV-eGFP-SARS-CoV-2 and rVSV-eGFP-EBOV. Both viruses formed plaques in Vero cells, however the sizes of plaques varied. rVSV-eGFP-SARS-CoV-2 formed rather small plaques at 72 h after infection, which could only be visualized under microscope magnification. In contrast, the plaques of rVSV-eGFP-EBOV were visible with naked eye (Figure 1F). This phenomenon suggested that the cytopathic effect of rVSV-eGFP-SARS-CoV-2 was much weaker than rVSV-eGFP-EBOV.

### Characterization of rVSV-eGFP-SARS-CoV-2 entry

**Figure 2.**
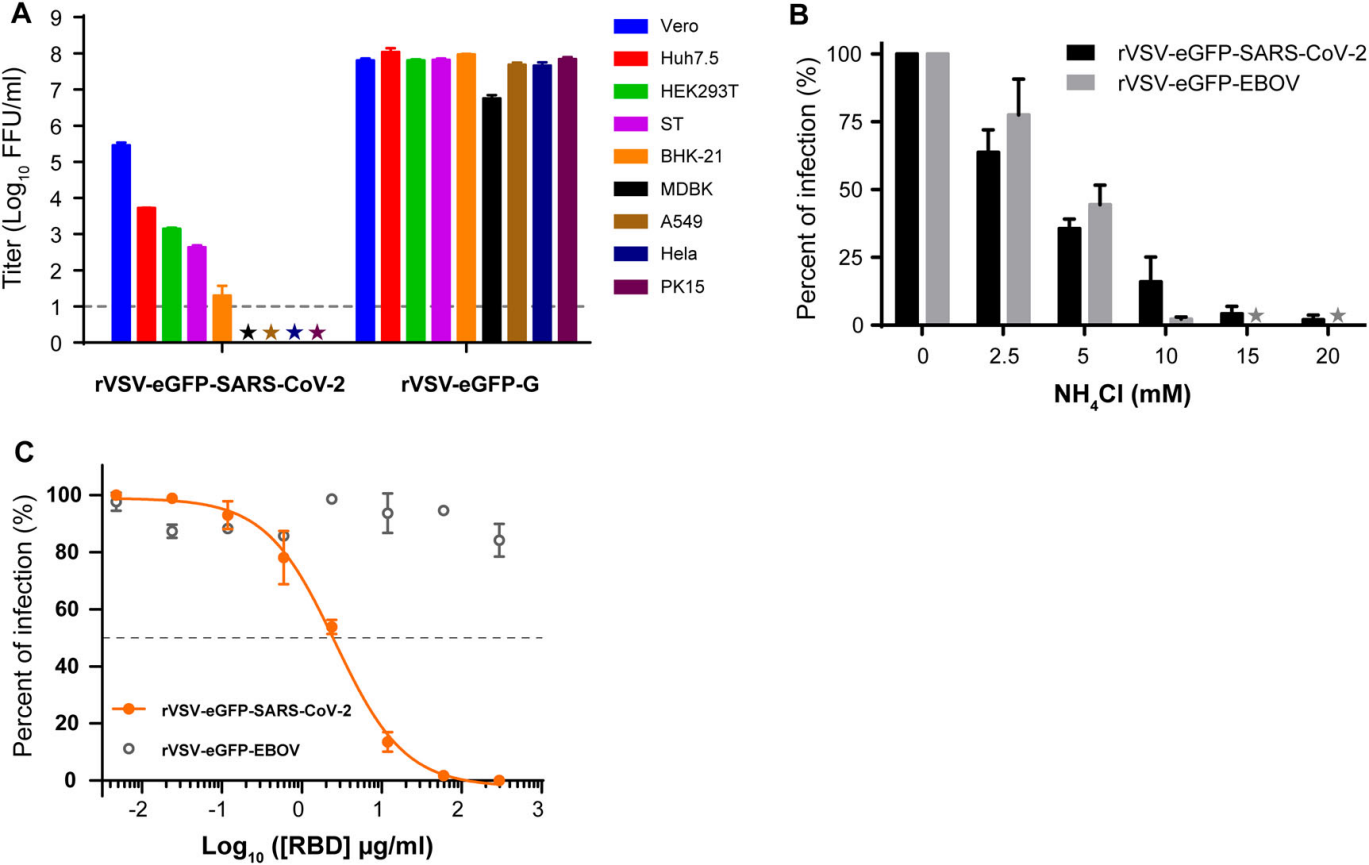
Characteristics of rVSV-eGFP-SARS-CoV-2 entry. (A) Infectivity of rVSV-eGFP-SARS-CoV-2 and rVSV-eGFP-G on various cell types. Infectivity was quantified by counting GFP positive cells in the above nine cell lines. The infectious titers were expressed as log_10_ FFU/ml. The limit of detection was 10 FFU/ml. Titers below 10 FFU/ml (★) are indicated. (B) Inhibition of rVSV-eGFP-SARS-CoV-2 infection by NH_4_Cl. Vero cells were infected with rVSV-eGFP-SARS-CoV-2 or rVSV-eGFP-EBOV at MOI of 0.01 after treatment with different concentrations of NH_4_Cl. The results are expressed as the percentage of the control (without NH_4_Cl treatment). (C) Effect of SARS-CoV-2 RBD fragment on the infectivity of rVSV-eGFP-SARS-CoV-2. All infection experiments were performed in triplicate. Error bars in (A–C) indicate standard deviation of the mean (n=3).

Replication-competent rVSV is a safe and convenient model for studying the entry process of SARS-CoV-2. We examined the cell tropism of rVSV-eGFP-SARS-CoV-2 using a panel of cell lines derived from hamster, swine, bovine, monkey, and human. Viral titers in the supernatant were measured by counting the GFP positive cell number 20 h post-infection. Among nine cell lines tested, four were susceptible to rVSV-eGFP-SARS-CoV-2 infection. Vero (African green monkey kidney cells) was most susceptible followed by Huh7.5 (human hepatocarcinoma cell line), HEK293T (human embryonic kidney cell line), and ST (swine testicle cells) (Figure 2A). BHK-21 (baby hamster kidney cell line), PK15 (porcine kidney cell line), Hela (human cervical adenocarcinoma cell line), A549 (human lung adenocarcinoma cell line), and MDBK (adult bovine kidney cell line) produced infections lower than the detection limit (10FFU/ml), thus were classified as resistant cell lines (Figure 2A). As expected, the positive control rVSV-eGFP-G infected all cell lines at similar levels (Figure 2A).

SARS-CoV-2 is internalized by receptor-mediated endocytosis and fused in the low-pH compartment [17]. To test whether rVSV-eGFP-SARS-CoV-2 entry is dependent on low pH, Vero cells were infected with rVSV-eGFP-SARS-CoV-2 or rVSV-eGFP-EBOV after treatment with concentrations of ammonium chloride (NH_4_Cl). NH_4_Cl is a lysosomotropic agent that can inhibit acidification of the endosomal compartment, thus preventing pH-dependent receptor-mediated endocytosis. Infection with rVSV-eGFP-EBOV, which can enter cells via pH-dependent endocytosis, was blocked by NH_4_Cl in a dose-dependent manner [18,19] (Figure 2B). Similarly, NH_4_Cl exposure also inhibited rVSV-eGFP-SARS-CoV-2 infection in Vero cells (Figure 2B). These results clearly indicate that rVSV-eGFP-SARS-CoV-2 entry is pH-dependent as in the wild-type virus.

The receptor binding domain (RBD) of the spike protein is proved to inhibit the entry of SARS-CoV-2 by binding to the ACE2 receptor on the plasma membrane [20]. We evaluated the effect of recombinant RBD fragment on rVSV-eGFP-SARS-CoV-2 entry. As shown in Figure 2C, RBD could abrogate rVSV-eGFP-SARS-CoV-2 entry in a dose-dependent manner with a 50% inhibitory concentration (IC_50_) of 2.6 µg/ml, whereas it had no inhibition effect for rVSV-eGFP-EBOV. These results suggest that rVSV-eGFP-SARS-CoV-2 mimics the entry process of SARS-CoV-2 and is suitable for entry studies.

### rVSV-eGFP-SARS-CoV-2 neutralization assay is highly correlative with pseudovirus and SARS-CoV-2

**Figure 3.**
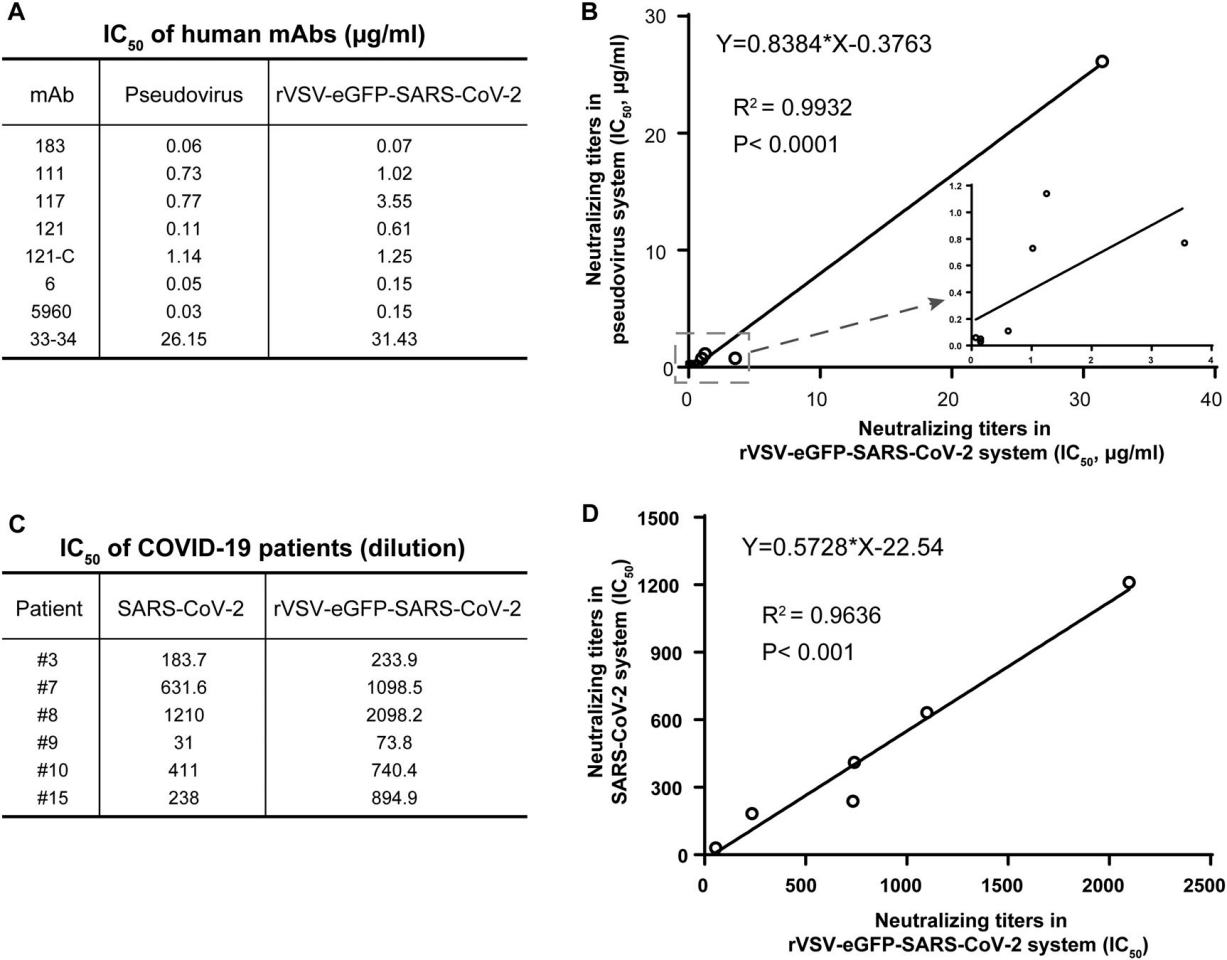
Comparison of rVSV-eGFP-SARS-CoV-2-based neutralization assay with pseudotype and live viruses. (A) IC_50_ values for 8 human mAbs against rVSV-eGFP-SARS-CoV-2 and VSV pseudotype. NAb titers of the mAbs against rVSV-eGFP-SARS-CoV-2 was evaluated by a focus reduction neutralization test. NAb titers against pseudovirus was evaluated by pseudovirus based neutralization assay (PBNA). IC_50_ was calculated by the Reed-Muench method. (B) Correlation of IC_50_ values in (A) between pseudovirus- and rVSV-based assays. (C) IC_50_ values for six serum samples from COVID-19 convalescent patients against rVSV-eGFP-SARS-CoV-2 and SARS-CoV-2. NAb titers of the serum samples against SARS-CoV-2 was determined by plaque reduction neutralization test, and IC_50_ was calculated by the Reed-Muench method. (D) Correlation of IC_50_ values in COVID-19 patients measured by rVSV-eGFP-SARS-CoV-2 and SARS-CoV-2.

A subset of eight human monoclonal antibodies (mAbs) was screened from single B cells of COVID-19 patients with neutralizing activity previously tested by the VSV-based pesudovirus system [16]. We determined their neutralizing titers against rVSV-eGFP-SARS-CoV-2. Three mAbs showed potent neutralizing activity with IC_50_ less than 200 ng/ml. Four mAbs exhibited mild neutralizing ability ranging from 0.61–3.55 μg/ml, while 33–34 had weak neutralizing activity (Figure 3A). We then compared the IC_50_ values obtained from our replication-competent system with VSV-based pseudotype viruses (Figure 3A). The NAb titers generated by the two systems exhibited a very strong correlation (R^2^=0.9932, *P*<0.0001) (Figure 3B), while the pseudovirus system seems more sensitive to neutralization than rVSV-eGFP- SARS-CoV-2.

We further tested the neutralizing activity of six serum samples from COVID-19 patients using both rVSV-eGFP-SARS-CoV-2 and live SARS-CoV-2. The serum samples were obtained from patients varying in illness severity. The IC_50_ of the sera ranged from 74 to 2098 for the rVSV and from 31 to 632 for the SARS-CoV-2 (Figure 3C). Although, the NAb titers against rVSV-eGFP-SARS-CoV-2 were higher than those against SARS-CoV-2, there was a high correlation between the two assays (R^2^=0.9636, *P*<0.001) (Figure 3D). Overall, these results strongly suggest that rVSV-eGFP-SARS-CoV-2 could be used as a surrogate model for SARS-CoV-2 neutralizing studies.

### Validation of the rVSV-eGFP-SARS-CoV-2-based neutralization assay

**Figure 4.**
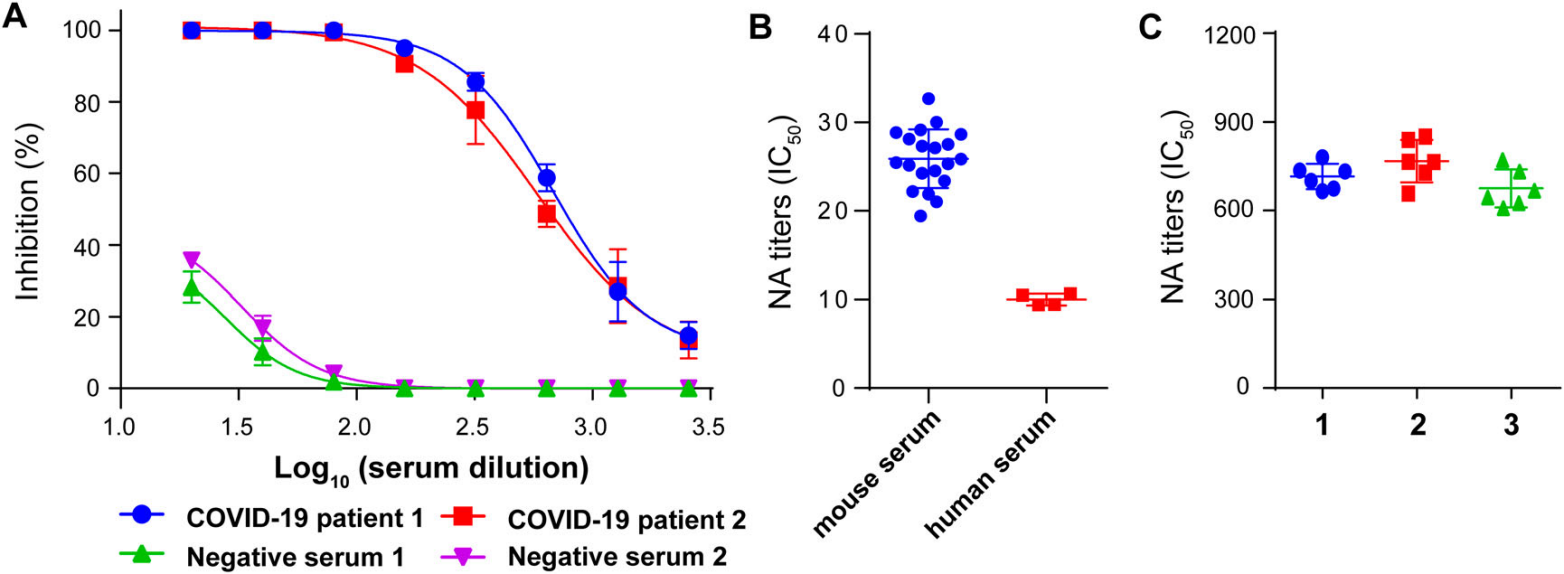
Validation of rVSV-eGFP-SARS-CoV-2-based neutralization assay. (A) Representative inhibition curves of two positive and two negative human serum samples. (B) The limit of detection (LOD) of the assay. A subset of negative serum samples from 20 mice and 4 humans was tested against rVSV-eGFP-SARS-CoV-2 using the focus reduction neutralization test. (C) Reproducibility of the neutralization assay. Pooled positive serum samples (n=2) were run six times per experiment in total of three experiments to measure reproducibility. Individual IC_50_ values were plotted to evaluate the intra- and inter-assay variability. Error bars in (A–C) indicate standard deviation of the mean.

Serum samples from two COVID-19 patients and two healthy donors were tested for the neutralizing activities against the rVSV-eGFP-SARS-CoV-2. Their typical neutralizing curves were shown as examples of positive and negative results (Figure 4A). To determine the limit of detection (LOD) of this assay, we measured the negative controls of 20 mouse and 4 human serum samples. LOD reflected the values at which positive results could be reliably detected. In this case, the LOD was 25.9 ± 3.3 (mean ± standard deviation) for mouse and 10 ± 0.7 for human serum samples (Figure 4B). Therefore, the cutoff of the neutralization assay was set as 1:30 for mouse and 1:10 for human serum samples separately. Sera with IC_50_ values above the LOD were considered as positive.

To evaluate the reproducibility of the rVSV-based neutralization assay, two positive human serum samples were pooled, and the neutralizing activities were tested against rVSV-eGFP-SARS-CoV-2. The neutralizing titers of the pooled sample were measured six times in one plate, in three separate experiments. The results from three experiments were plotted and compared (Figure 4C). The coefficient of variation of both intra-assay and inter-assay was less than 10%, indicating acceptable reproducibility. Thus, we validated the rVSV-based neutralization assay with good specificity and reproducibility.

## Discussion

SARS-CoV-2 is highly infectious and causes a life-threatening disease. One of the greatest limitations for SARS-CoV-2 study is the requirement of the BSL3 laboratory to manipulate live virus. Pseudotype viruses bearing heterologous viral glycoproteins can be powerful tools for studying the entry of many viral pathogens, including SARS-CoV [21,22]. SARS-CoV-2 pseudotype viruses based on lentivirus or VSV backbones have been established [14,17] and used for detection of the neutralizing activity of human sera and antibodies [7,16,23]. However, the production of pseudotype viruses requires plasmid purification and transfection, which is time and labor intensive. Furthermore, the batch variation of pseudoviruses limits their applications in large-scale screening and clinical evaluation of potential vaccines. In this study, we generated an infectious recombinant VSV, which could effectively package SARS-CoV-2 S protein into the viral particles. The rVSV-eGFP- SARS-CoV-2 displayed high infectivity in susceptible cells, and viral infection could be scored using GFP-expressing cells. Since rVSV-eGFP-SARS-CoV-2 is able to propagate, repeated cell culture and transfection are no longer needed, which saves time and effort. Furthermore, the multiple infection features of rVSVs better imitate the native SARS-CoV-2 life cycle; the results generated using rVSVs could be similar to those of SARS-CoV-2. Notably, rVSV-eGFP-SARS-CoV-2 infection can be quantified and analysed by a high-content imaging system, as shown in Figure S1, illustrating an automated assay format. We also rescued the rVSV-luc-SARS-CoV-2 encoding luciferase reporter. The luciferase signals can be measured by luminescence plate readers in high-throughput format.

Packaging the heterogenous envelope proteins onto rVSVs typically involves modification of the transmembrane domain or the intracellular domain. Deletion of the ER-retention signal in the intracellular domain of S significantly increases the packaging efficiency of coronaviruses, such as SARS-CoV and PEDV [22,24–27]. However, in our design, the wild-type S was efficiently incorporated into rVSV particles with a titer of around 10^6^ FFU/ml, which is sufficient for an entry assay.

Based on our cell tropism analysis, two of four human cell lines (Huh7.5 and HEK293T) were highly susceptible to rVSV-SARS-CoV-2. Among five non-human cell lines, cells from monkey (Vero) and pig (ST) were susceptible, but cells from hamster (BHK-21), bovine (MDBK), and another porcine cell line (PK15) were resistant. These results were generally consistent with cell tropism profiles determined by wild type SARS-CoV-2, except for PK15 [28–30]. Chu et al. reported that SARS-CoV-2 could infect and replicate efficiently in PK15 cells measured by quantitative RT–PCR [29]. However, Conceicao et al. demonstrated a very low level of SARS-CoV-2 infection in PK15 cells even at a higher MOI [30]. The controversy about the susceptibility of PK15 cells was likely due to differences in testing methods.

The NAb titers generated against rVSV-eGFP-SARS-CoV-2 demonstrated a strong correlation with those against pseudovirus as well as SARS-CoV-2. In addition, we noticed that the sensitivity of rVSV-based neutralization assay was lower than the pseudovirus system, but higher than the wild type virus. This might be due to different readout of neutralization methods.

In conclusion, we describe a replication-competent rVSV carrying the SARS-CoV-2 S protein as a surrogate model for studying SARS-CoV-2. This model is safer, more consistent, and convenient compared with available alternative approaches. Therefore, rVSVs possess great potential in studying virus-host interactions, characterizing immune responses, screening and detecting neutralizing antibodies, and developing novel therapeutics.

## Supplementary Material

Fig_S1-clean.docx
