# Supplementary material for: Establishment of replication-competent vesicular stomatitis virus-based recombinant viruses suitable for SARS-CoV-2 entry and neutralization assays
Source: Emerg Microbes Infect. 2020 Oct 17;9(1):2269–77. doi: 10.1080/22221751.2020.1830715 (PMC7594855; doi:10.1080/22221751.2020.1830715)
Supplement: Fig_S1-clean.docx [file TEMI_A_1830715_SM0876.docx]

**Supplementary information**

**Fig. S1. Automated format of neutralizing assay using high content image systems.** (A) Application of high content image system to determine the neutralizing activities of two human mAbs against rVSV-eGFP-SARS-CoV-2. The fluorescence images were acquired 20 h after infection. Each concentration is shown in duplicate. (B) Average values of GFP positive cell number in duplicate wells were quantified and plotted with a non-linear regression curve using GraphPad Prism. Gray dashed line indicates 50% inhibition. The IC_50_ of both antibodies were calculated by the Reed-Muench method.
